# Demographic and Clinical Correlates of Quality of Life Domains in Spinal Cord Injury

**DOI:** 10.3390/healthcare14030357

**Published:** 2026-01-30

**Authors:** Monika Zackova, Paola Rucci, Golcin Maknouni, Simona Udriste, Emanuele Salvatori, Maria Cristina Pirazzoli

**Affiliations:** 1 Montecatone Rehabilitation Institute, 40026 Imola, Italy; golcin.maknouni@montecatone.com (G.M.); danasimona.udriste@montecatone.com (S.U.); emanuele.salvatori@montecatone.com (E.S.); cristina.pirazzoli@montecatone.com (M.C.P.); 2 Department of Biomedical and Neuromotor Sciences, University of Bologna, 40138 Bologna, Italy; paola.rucci2@unibo.it

**Keywords:** spinal cord injury (SCI), quality of life (QoL), rehabilitation, WHOQOL-BREF

## Abstract

**Highlights:**

**What are the main findings?**

- Age, gender, and living situation shape quality of life after spinal cord injury.
- Social and psychological well-being are particularly affected in older adults and those living alone.

**What are the implications of the main findings?**

- Regular Quality of Life assessments can guide personalized rehab plans.
- Tailored interventions targeting vulnerable groups may boost recovery and community reintegration.

**Abstract:**

**Background/Objectives**: In patients with spinal cord injury (SCI), quality of life (QoL) is increasingly recognized as an important indicator of their ability to sustain both the rehabilitation process and post-rehabilitation community reintegration, and it plays a crucial role in prognosis. The primary purpose of this study was to identify the demographic and clinical correlates of different QoL domains as perceived by patients hospitalized after SCI. **Methods**: We conducted a cross-sectional study on a single day in the units of the Montecatone Rehabilitation Institute (MRI), the largest Italian center for intensive rehabilitation of individuals with SCI. We administered the World Health Organization Quality of Life–Short Version (WHOQOL-BREF), which consists of 26 items rated on a five-point Likert scale. Study participants included 88 adults with SCI; a total of 74% were male, with a mean age of 53.3 years (SD = 15.05). The lesion was traumatic in 74% of cases and complete in 59%. Physical health showed a weak negative association with age (r = −0.213, *p* = 0.05), whereas social QoL demonstrated a significant positive association with age (r = 0.215, *p* = 0.046). Psychological QoL was significantly lower in females compared with males (46.9 vs. 55.1, *p* < 0.05) and in patients living alone compared with those not living alone (46.1 vs. 54.6, *p* < 0.05). **Conclusions**: Clinicians should consider routine assessment of QoL to personalize post-discharge therapeutic plans and to implement targeted interventions aimed at improving outcomes in patients with SCI.

## 1. Introduction

Spinal cord injury (SCI) is a severe condition with a devastating impact, involving profound biological, psychological, and social changes in affected individuals. Its prevalence and incidence can vary depending on definitions, data sources, and reporting systems. In the world, 20.6 million (95% UI 18.9 to 23.6) individuals were living with SCI in 2019. The incidence of SCI was 0.9 million (0.7 to 1.2) cases. SCI rates increased substantially from 1990 to 2019 for global prevalence (81.5%, 74.2 to 87.1), incidence (52.7%, 30.3 to 69.8) [1].

Across Europe, the prevalence of SCI has been estimated between 250 and 900 per million inhabitants. This wide range reflects differences in healthcare systems, registry quality, and population age structures [2].

The incidence of traumatic SCI in Europe ranges between 10 and 40 per million per year. Higher rates are reported in countries with more road traffic and extreme sports participation, while lower rates are noted in Northern Europe (e.g., Scandinavia).

Notably, about 70–80% of SCI patients are male and the mean age at injury is rising—now typically around 40–50 years, reflecting aging populations [3,4].

Italy is estimated to have between 20,000 and 30,000 people living with long-term spinal cord injuries. Estimates vary, but the prevalence rate is around 400–500 per million inhabitants. As to the incidence, each year, Italy reports about 1500–2000 new cases of traumatic SCI, which chis corresponds to an incidence rate of approximately 20–30 new cases per million people per year [5]. The majority of traumatic SCIs in Italy occur in young and middle-aged males, often due to road traffic accidents, falls, and sports injuries [6]. Non-traumatic SCI (e.g., due to tumors, infections, or vascular causes) adds an additional burden, possibly equal to or greater than traumatic cases in older populations [7].

Due to the largely irreversible nature of SCI, current therapeutic strategies cannot achieve complete neurological recovery. Consequently, clinical management focuses primarily on preventing secondary deterioration rather than promoting neural repair. As a result, many patients are left with permanent disabilities that substantially affect both physical and psychological health. Given the limitations of available therapeutic options, comprehensive multidisciplinary rehabilitation—including physical, occupational, and psychological therapy, as well as the use of assistive devices—is essential to promote the highest possible level of autonomy and to enhance quality of life (QoL). Therefore, primary prevention remains the most critical strategy for addressing SCI.

In this context of widespread suffering, patients’ quality of life is increasingly considered an indicator of their ability to sustain both the rehabilitation process and post-rehabilitation community reintegration, as well as an outcome measure of the quality of care provided.

The World Health Organization (WHO) defines quality of life as an individual’s perception of their position in life within the context of the culture and value systems in which they live, as well as in relation to their goals, expectations, standards, and concerns [8]. Quality of life is a multidimensional construct comprising four domains: physical health, psychological health, social relationships, and environment.

The quality of life with SCI depends mostly on the availability of appropriate resources and services, the presence of supportive relationships and community inclusion, and on whether people have to confront discriminatory attitudes and other obstacles. The overall impact of SCI on the individual, and also on society at large, therefore depends on a range of factors, including: the age at which the injury occurs; the extent and the characteristics of the injury; the availability and timing of resources and services; the environment in which the person lives—including physical, social, economic and attitudinal factors [9].

In the present study, we used the World Health Organization Quality of Life–Short Version (WHOQOL-BREF), which assesses these domains with reference to the preceding two weeks. The WHOQOL-BREF has been identified as one of the most acceptable and well-established instruments for assessing QoL after SCI in a systematic review [10].

However, evidence regarding the relationship between QoL and demographic and clinical variables remains inconsistent across studies and varies according to recruitment setting, illness phase, and geographical area.

The primary purpose of this study was to address this knowledge gap by providing evidence on the demographic and clinical correlates of different QoL domains as perceived by patients hospitalized after spinal cord injury. We hypothesized that age, sex, living situation, and injury characteristics would be significantly associated with differences in QoL domains among hospitalized SCI patients. The paper is organized as follows: Section 2 reports the study design, the procedures for data collection, the QoL assessment and the statistical analysis. Section 3 reports the characteristics of the sample, the relationship of QoL with demographic and clinical characteristics, the pattern of individual responses and patients’ qualitative feedback. Lastly, in the Section 4 we compare the study results with evidence from the literature and report on the strengths and limitations of the study, and the future perspectives.

## 2. Materials and Methods

### 2.1. Study Design and Setting

A cross-sectional survey was conducted on a single day in the units of the Montecatone Rehabilitation Institute (MRI), which is the largest Italian center for intensive rehabilitation of individuals with spinal cord injury and a center of excellence for acquired brain injury. MRI is also the only facility in the European Union with an intensive care unit dedicated to spinal cord and brain injuries. The institute has a total of 150 beds and includes an intensive care unit, a day hospital, and a Spinal Unit–Return, a nurse-managed unit dedicated to individuals with chronic SCI and/or brain injury who require specialized treatments following the onset of complications (e.g., pressure injuries, neurogenic bowel and bladder dysfunction, spasticity, neuropathic pain).

### 2.2. Participants and Eligibility Criteria

On the day of the survey, all hospitalized patients of both sexes, with SCI at any neurological level and of any etiology or completeness were eligible for inclusion, provided they had sufficient Italian proficiency to complete the questionnaires and gave informed consent after receiving an explanation of the study procedures. Exclusion criteria included cognitive impairment and the presence of brain injury.

### 2.3. Data Collection and Variables

Clinical and demographic variables were extracted from medical records or collected via interview and linked with questionnaire data. These variables included age, sex, educational level, marital status, living condition, employment status, etiology, neurological impairment classification according to the American Spinal Injury Association (ASIA) definitions, comorbidities, time since injury, length of hospital stay, and clinical status (acute or chronic). The WHOQOL-BREF questionnaire was administered in paper-and-pencil format and completed independently by patients or with the assistance of health professionals not involved in their care.

### 2.4. Quality of Life Assessment (WHOQOL-BREF)

The WHOQOL-BREF consists of 26 items rated on a five-point Likert scale, with higher scores indicating more positive perceptions. Response options vary by item and assess either satisfaction or frequency of experiences. Three items (physical pain, need for medical treatment, and negative feelings) are negatively phrased and are reverse-scored by subtracting the item score from six. Three additional optional questions address time taken to complete the questionnaire, need for assistance, and open comments.

The WHOQOL-BREF has been used to characterize patients with SCI in relation to spasticity severity [11], etiology and duration of injury [12], injury completeness, and neurological level [13]. Additionally, it has been employed to analyze associations between functional independence, QoL, sitting balance, and wheelchair ability in full-time wheelchair users with SCI [14], as well as predictors of QoL following discharge from hospital to the community in individuals with paraplegic SCI [15].

The Italian version of the WHOQOL-BREF was validated by De Girolamo et al. [16]. In patients with traumatic SCI, the instrument has demonstrated good internal consistency across all domains (Cronbach’s alpha range: 0.74–0.78), except for social relationships (alpha = 0.54), and good discriminant validity [17].

### 2.5. Statistical Analysis

A sample size of at least 79 patients was needed for a multiple regression analysis with an effect size f^2^ = 0.08, a power of 80%, alpha = 0.05 and 11 predictors. Categorical variables were summarized using absolute and percentage frequencies, while continuous variables were described using means and standard deviations or medians and interquartile ranges, as appropriate. Univariate normality of the WHOQOL-BREF domain distribution was tested using the Shapiro–Wilk test and multivariate normality using the Henze-Zirkler test [18]. As no departure from normality was found, WHOQOL-BREF domain scores were compared between groups using *t*-tests or analysis of variance. Homogeneity of variance was tested using Levene’s test. Item scores were compared within the overall sample using paired-sample *t*-tests. Individual response profiles were graphically represented as percentages of items rated as 4 (good/satisfactory) or 5 (very good/very satisfactory) overall ratings.

Pearson’s and Spearman’s correlation coefficients, as well as linear and non-linear regression models, were used to examine associations between domain scores and continuous variables. The probability to enter in stepwise regression models was set to *p* = 0.05 and that for exit to *p* = 0.10. Collinearity was tested using tolerance. Regression standardized residuals were analysed using the normal probability plot and Durbin-Watson statistics. The significance level was set to *p* < 0.05. A Bonferroni correction for multiple comparisons was applied to the probability level. The sample size calculation was performed using G*Power 3.1.9.4, Copyright 1992–2019. All the statistical analyses were conducted using IBM SPSS, Version 30.0, IBM Corporation (Armonk, NY, USA), 1989, 2024.

### 2.6. Ethical Considerations

The study was conducted in compliance with the Declaration of Helsinki (DoH)—Ethical Principles for Medical Research Involving Human Participants (1964) and its latest amendments adopted by the 75th General Assembly of the World Medical Association (WMA) in Finland on 19 October 2024.

## 3. Results

### 3.1. Characteristics of the Study Sample

Of the 94 patients who participated in the study, 88 were included in the analyses and 6 were excluded because they failed to complete at least 21 WHOQOL-BREF items.

A listwise deletion of the 6 missing cases was performed in all analyses. The final sample had a mean age of 53 years (SD = 15.05) and was predominantly male (74%). The median questionnaire completion time was 10 min (IQR: 5–15 min); 41.7% of participants completed it independently, while 58.3% required assistance. Patients requiring assistance had poorer physical (47.6 ± 10.2 vs. 52.2 ± 10.7, *t*-test = 1.9, *p* = 0.03) and social QoL (58.5 ± 19.0 vs. 66.8 ± 17.7, *t*-test = 2.02, *p* = 0.023) compared with the rest of the sample, but similar QoL levels in the psychological and environmental domains.

Table 1 summarizes the sociodemographic and lesion characteristics of the sample. Most injuries were traumatic in etiology, and 59% of patients had a complete lesion (AIS grade A). The male-to-female ratio was 3.64:1 in traumatic injuries and 1.87:1 in non-traumatic injuries. The median time since injury was six months

**Figure 1:**
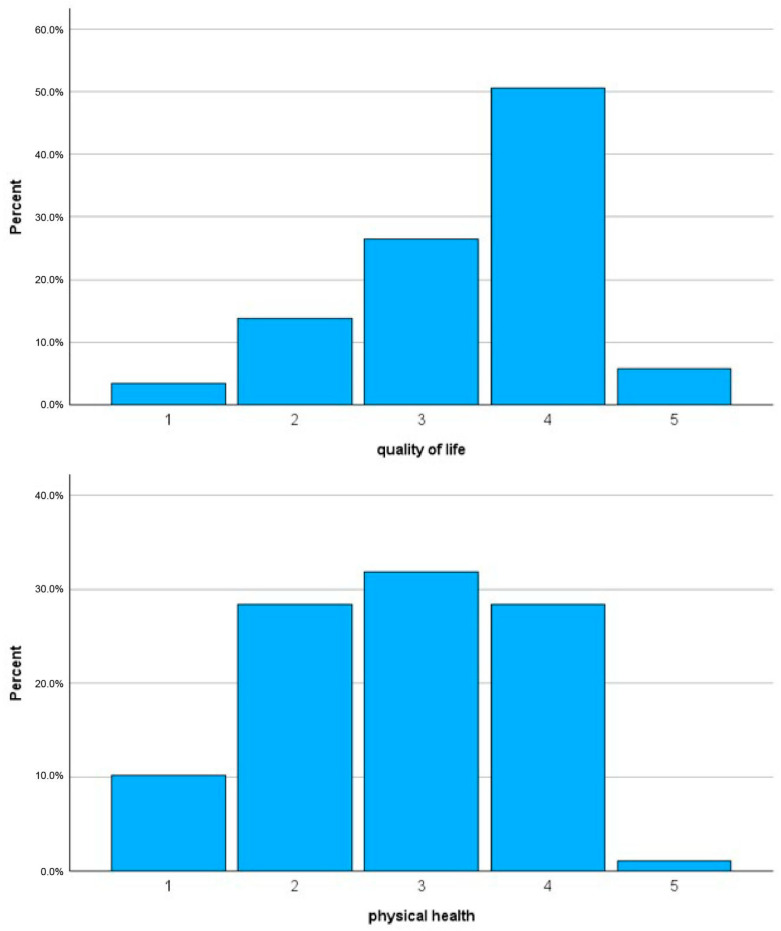
Patients’ ratings of quality of life (1 = very poor, 2 = poor, 3 = neither poor nor good; 4 = good, 5 = very good) and physical health (1 = very dissatisfied, 2 = dissatisfied, 3 = neither satisfied nor dissatisfied, 4 = satisfied, 5 = very satisfied).

Figure 1 illustrates overall ratings of quality of life (item 1) and physical health (item 2). The mean QoL rating was significantly higher than that for physical health (3.41 ± 0.92 vs. 2.80 ± 1.00; *t* = 6.1, *p* < 0.01).

Table 2 presents WHOQOL-BREF domain scores for the overall sample. Physical health had the lowest mean score, whereas social relationships showed the highest mean score and greatest variability.

### 3.2. Quality of Life and Demographic Characteristics

**Figure 2:**
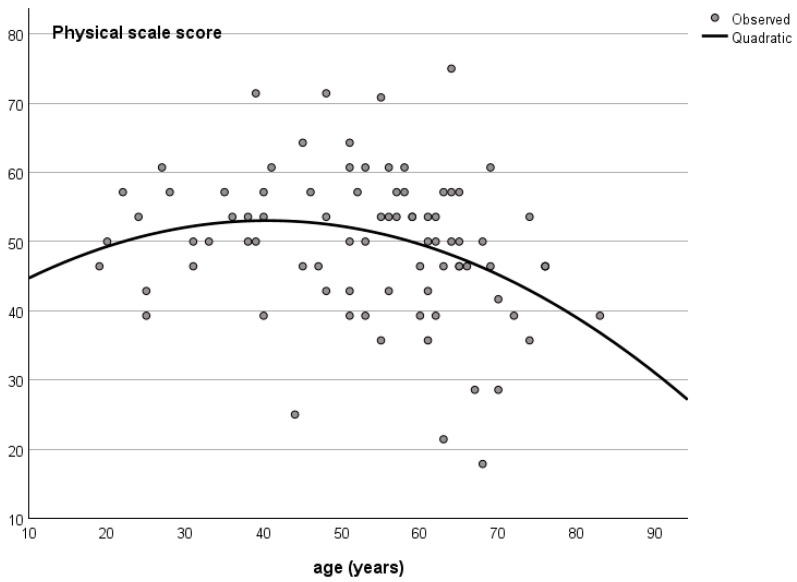
Relationship between age and WHOQOL-BREF physical scale score.

Physical health showed a weak negative association with age (r = −0.213, *p* = 0.05), while social QoL demonstrated a significant positive association with age (r = 0.215, *p* = 0.046). Age was not significantly related to psychological QoL (r = −0.179, *p* = 0.097) or the environment domain (r = −0.023, *p* = 0.834). Non-linear regression analysis indicated a quadratic relationship between age and physical QoL, with improvement up to approximately 40 years followed by a decline thereafter (Figure 2). The model diagnostic did not reveal departure from normality in the residuals.

Psychological QoL was significantly lower in females than in males (46.9 vs. 55.1, *p* < 0.05) and in patients living alone compared with those not living alone (46.1 vs. 54.6, *p* < 0.05). Environmental QoL was lower among patients with a lower educational level (54.1 vs. 60.9). No significant associations were observed between QoL domains and marital or employment status.

### 3.3. Quality of Life and Lesion Characteristics

Among clinical variables, only psychological QoL varied significantly according to injury characteristics. Patients with non-traumatic etiology (45.9 vs. 55.3, *p* = 0.007), AIS grade B injuries (48.9 vs. 55.9, *p* < 0.05), and thoracic neurological levels (48.6 vs. 56.4, *p* = 0.007) reported significantly lower psychological QoL than their counterparts. However, in a multiple stepwise linear regression model including age, sex, living condition, etiology, neurological level, and AIS grade, only female sex (β = −0.233), living alone (β = −0.259), and non-cervical neurological level (thoracic or lumbar) (β = −0.336) significantly predicted lower psychological QoL (Table 3). Neurological level showed the strongest effect, and the model explained 24.9% of the variance. The diagnostics of the model did not reveal multicollinearity in the included variables, autocorrelation in the residuals, outliers or skewness in the data.

Time from injury was negatively associated with social QoL (Spearman’s rho = −0.286, *p* < 0.01), indicating poorer social relationships with longer post-injury duration (Table 4). Length of hospital stay was not significantly associated with any QoL domain (Spearman’s rho ranged from −0.013 for physical health to −0.195 for psychological health).

### 3.4. Individual Responses and Qualitative Feedback

**Figure 3:**
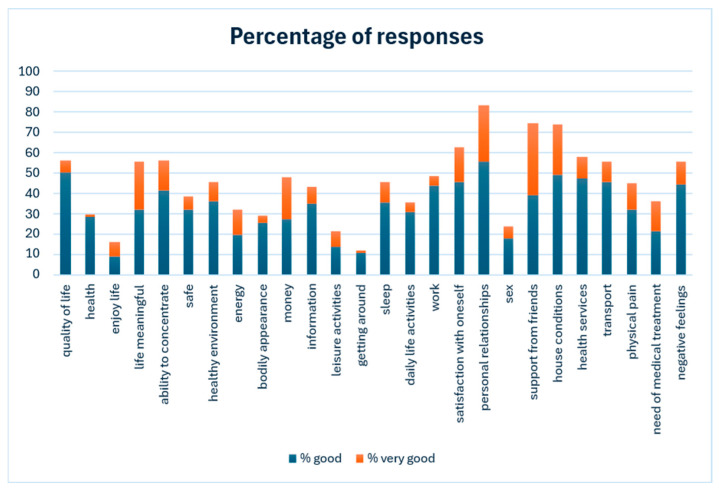
Percentage of WHOQOL-BREF items rated as good or very good.

Analysis of individual item responses (Figure 3) revealed that a high proportion of patients rated interpersonal relationships, support from friends, and housing conditions as satisfactory or very satisfactory. In contrast, only a small minority rated their health, mobility, enjoyment of life, and sexual activity positively. Forty-six patients (52.3%) responded to the open-ended question; 35 had no comments, while 11 considered the WHOQOL-BREF items too generic or irrelevant to their specific health conditions.

## 4. Discussion

### 4.1. Main Findings and Comparisons with the Literature

Our results indicate that female sex, living alone, and a non-cervical level of injury were associated with lower psychological QoL. Physical quality of life (QoL) increased up to around the age of 45 years, after which it gradually declined. Additionally, a longer time since injury was associated with poorer social relationships.

The findings of our study indicate that individuals with SCI are highly vulnerable and face difficulties in meeting their social, occupational, health, and personal needs, consistent with studies conducted in other countries [19,20]. Our study was carried out in a tertiary national excellence center for SCI, receiving patients from across Italy, and thus can be considered representative of different geographical areas. Patients generally had a high level of education and significant clinical complexity (AIS-A or B), with the majority of injuries of traumatic etiology. Compared with community-dwelling SCI patients [21], our sample demonstrated markedly reduced QoL across all domains. The high percentage of patients with traumatic injury and male-to-female ratio in the subgroups with traumatic and non-traumatic injury are consistent with the epidemiology of patients with SCI treated in a hospital setting [22,23].

Regarding the relationship between QoL and demographic characteristics, age showed a non-linear association with physical QoL, with a hinge point at 40 years. Previous studies have reported that older age is associated with worse QoL outcomes [24], and non-linear relationships have also been observed, with middle age (30–59 years) linked to pain interference [25], and age greater than 35 has been associated with unsatisfactory coping [26].

In our study, psychological QoL varied significantly, being lower in females. Gender differences in psychological QoL are common across many physical and mental health conditions and may reflect societal role expectations or differences in coping strategies among males and females with SCI. A large study comparing the SCI experience by gender in 22 countries found that females were more likely to report higher satisfaction with services and lower involvement in decision-making [27].

Although psychological quality of life did not differ between married and unmarried patients, it was lower in individuals living alone. Other studies have reported higher QoL in unmarried patients [28]. Our findings mirror the evidence from a review underscoring the social disconnectedness and perceived social isolation of people with SCI [29].

Patients with lower educational attainment had reduced QoL in the environment domain, likely reflecting limited access to healthcare resources and reduced ability to acquire the information needed to manage the complex demands of SCI. We found no significant relationship between employment status and QoL. However, previous studies have linked unemployment to lower life satisfaction [20,25], suggesting that the impact of vocational engagement may vary across healthcare systems and the availability of social support.

Injury severity, including completeness, etiology, and neurological level, was negatively associated with psychological QoL, but only neurological level remained significant in a multiple regression analysis. Patients with non-cervical injuries had lower psychological QoL. Longer post-injury duration was associated with poorer social QoL. On the contrary, Tzanos et al. [20] reported a positive correlation between time since injury and the environment domain of WHOQOL, and Geyh et al. [19] found that longer time since injury was linked to higher overall QoL. A possible explanation of this difference is that in a hospital setting (as in our study), long-term patients may present with secondary complications that explain this decline, unlike those successfully reintegrated into the community. These discrepancies may also reflect differences in rehabilitation approaches, cultural contexts, or social support availability across countries.

Analysis of individual responses revealed that interpersonal relationships and support from friends are relative strengths for SCI patients. When the immediate family is absent, care is typically provided by immediate family members or close relatives [30]. Therefore, the prevention, management, and long-term care of SCI survivors should be planned within a social-ecological framework [31,32]. However, many SCI survivors lack available family support, and approximately 69% are partially or fully dependent on caregivers. This highlights the need for alternative long-term care strategies.

Patients with spinal cord injuries often exhibit vulnerability in psychological, spiritual, and family support and face challenges in meeting their social, occupational, health, and personal needs. Individuals who experience the psychological trauma of SCI are at increased risk for mental health problems [33,34,35]. In our study, participants requiring partial assistance with activities of daily living (ADL) often could not achieve full function due to poor psychological health. This reliance on family care has been observed in many countries, including Italy [18].

The findings of our study emphasize the importance of encouraging people with spinal cord injuries (SCI) to play an active role in their healthcare, especially after they are discharged. Providing structured information and training during rehabilitation as well as involving family members in this education, is essential to ensuring continuity of care and adherence to treatment plans. Supporting family members and other caregivers is also crucial, as it can prevent stress, burnout, and fatigue.

In settings with sufficient resources, an independent living model with personal assistance can empower individuals with SCI who have high support needs and be cost-effective. In regions with limited resources, community-based rehabilitation (CBR) programs play a vital role in promoting participation, social inclusion, and empowerment. Social networks, peer support groups, and disabled people’s organizations are valuable across all settings in fostering self-efficacy, participation, and psychological well-being.

Access to physical activity and sports should be encouraged as these activities promote physiological and psychological health. To facilitate vocational integration, it is recommended that supported employment, vocational training, and flexible working arrangements are in place, while ensuring that social protection schemes are available without discouraging a return to work.

Together, these strategies support a holistic approach to SCI care that addresses medical, psychological, social, and occupational needs and aligns with international perspectives on best practices for SCI care. [9].

### 4.2. Strengths

The study has several strengths. Firstly, it was conducted in a national referral center, thereby ensuring variability among patients. Secondly, it was based on a multidimensional quality of life (QoL) assessment that captured the nuances of patients’ experiences, as well as a multivariable analysis of the potential predictors of QoL.

### 4.3. Limitations of the Study and Future Directions

Our sample size was relatively small, limiting our ability to detect differences across multiple demographic and clinical variables. Although the WHOQOL-BREF is widely accepted as a measure of QoL after SCI, it is a generic instrument intended for the general population. Some participants reported that certain questions were irrelevant to SCI patients. Future studies would benefit from the development of QoL instruments specifically tailored to this population. There is a potential bias related to the fact that more than half of the patients completed the questionnaire with the help of a healthcare professional. While this raises concerns about social desirability or proxy interpretation, the comparison of QoL scores between patients who required assistance and those who completed the questionnaires independently highlighted poorer physical and social quality of life in the former group. This finding is consistent with the expectation that patients who ask for help will have lower scores in domains relating to the ability to perform daily tasks, thereby mitigating the risk of bias. Furthermore, our results should be generalized with caution, as patients treated in a tertiary hospital setting may differ from those treated in less specialized facilities or in the community.

## 5. Conclusions

For hospitalized patients with spinal cord injury, physical functioning was the most affected quality of life domain. A lower psychological QoL was associated with being female, living alone and having a non-cervical injury. Patients reported high satisfaction with interpersonal relationships and support from friends. Future research should explore targeted interventions to improve outcomes for these groups by leveraging their social networks.

## Figures and Tables

**Table 1:** Demographic and clinical characteristics of the study sample (N = 88).

Patient Characteristics	
Age, mean ± SD	53.2 ± 15.0
Male, n (%)	66 (75%)
Educational level *	
Primary school	4 (5%)
Secondary school	21 (24%)
High school or higher	62 (71%)
Living condition *	
Alone	18 (21%)
With others	69 (79%)
Married/living with partner	49 (56%)
Currently working	41 (47%)
Etiology, n (%)	
Non-traumatic	23 (26%)
Traumatic	65 (74%)
Neurological level, n (%)	
Cervical	52 (59%)
Thoracic	34 (39%)
Lumbar	2 (2%)
Any comorbid condition	62 (71%)
AIS grade	
A	52 (59%)
B	35 (40%)
C	1 (1%)
Length of hospital stay (days), median (IQR)	56 (32–106)
Time from the lesions (months), median (IQR)	6.5 (3.3–60)
Current status	
Acute	48 (55%)
Chronic	40 (45%)

* Information was not disclosed by one participant in the self-report form. Abbreviations: AIS American Spinal Injury Association Impairment Scale grade, IQR interquartile range.

**Table 2:** WHOQOL-BREF domain scores by demographic and clinical characteristics. Data are expressed as mean ± standard deviation.

	WHOQOL-BREF Domains							
	Physical		Psychological		Social		Environment	
Overall sample	49.6 ± 10.5		52.8 ± 14.5		62.2 ± 18.8		58.9 ± 14.0	
Sex								
Female	49.5 ± 8.7		46.9 ± 13.9 *		59.3 ± 18.2		59.8 ± 11.9	
Male	50.2 ± 11.0		55.1 ± 14.6		62.9 ± 19.8		58.6 ± 14.8	
Single								
No	49.7 ± 11.2		53.6 ± 13.9		63.5 ± 21.2		59.1 ± 13.8	
Yes	49.7 ± 9.9		51.8 ± 15.6		60.5 ± 15.6		58.4 ± 14.5	
Living situation								
Alone	46.8 ± 11.2		46.1 ± 16.1 *		62.0 ± 17.0		57.5 ± 16.5	
With others	50.5 ± 10.4		54.6 ± 13.8		62.2 ± 19.5		59.1 ± 13.4	
Educational level								
Less than high school	48.1 ± 11.4		53.2 ± 15.4		61.3 ± 21.2		54.1 ± 16.8 *	
High school or other	50.4 ± 10.2		52.5 ± 14.3		62.2 ± 17.8		60.9 ± 12.5	
Working condition								
Employed	49.5 ± 11.3		54.1 ± 14.9		62.7 ± 15.8		60.5 ± 14.7	
Not working	49.5 ± 10.1		51.9 ± 14.5		62.4 ± 21.4		58.7 ± 13.0	
AIS grade								
A	50.7 ± 10.3		55.9 ± 14.5		65.1 ± 17.5		61.5 ± 14.5	
B	48.2 ± 10.9		49.0 ± 13.5 *		58.3 ± 20.2		55.7 ± 12.6	
C °	N/A	N/A	33.3	N/A	50.0	N/A	40.6	N/A
Neurological level of injury								
Cervical	51.2 ± 8.4		56.4 ± 13.5		64.6 ± 16.8		61.0 ± 12.6	
Thoracic	48.1 ± 12.3		48.7 ± 14.5 *		59.3 ± 21.6		56.2 ± 16.0	
Lumbar	35.7 ± 20.2		33.3 ± 11.8		50.0 ±		51.6 ± 2.2	
Etiology								
Traumatic	49.8 ± 11.0		55.3 ± 14.7		62.7 ± 20.1		60.2 ± 14.2	
Non-Traumatic	49.1 ± 9.1		45.9 ± 11.8 **		60.9 ± 15.0		55.6 ± 13.2	
Comorbidity								
Yes	48.8 ± 11.5		52.2 ± 14.8		64.0 ± 18.4		59.4 ± 14.6	
No	51.6 ± 7.5		54.4 ± 14.0		57.7 ± 19.4		57.9 ± 12.8	
Current status								
Acute	49.7 ± 10.3		51.7 ± 14.6		64.5 ± 18.5		56.9 ± 14.7	
Chronic	49.5 ± 10.9		54.1 ± 14.5		59.6 ± 18.9		61.3 ± 13.1	

* *t*-test is significant at *p* < 0.05, ** *t*-test is significant at *p* < 0.01. ° This category includes just one patient, so the standard deviation is not reported. The physical domain was not computed because of missing items. Abbreviations: AIS, American Spinal Injury Association Impairment Scale.

**Table 3:** Clinical predictors of psychological QOL in a stepwise multiple linear regression model.

Model				Standardized Coefficients	*t*	Sig.	95.0% CI for b	
		B	SE(b)	Beta			Lower Bound	Upper Bound
1	(Constant)	47.574	2.424		19.630	<0.001	42.753	52.394
	Non-cervical level of injury	−8.783	3.129	−0.294	−2.807	0.006	−2.560	−15.006
2	(Constant)	49.230	2.431		20.248	<0.001	44.394	54.067
	Non-cervical level of injury	−9.335	3.035	−0.313	−3.076	0.003	−3.298	−15.372
	Living_alone	−9.389	3.639	−0.263	−2.580	0.012	−16.628	−2.150
3	(Constant)	43.043	3.547		12.135	<0.001	35.986	50.100
	Non-cervical level of injury	−10.012	2.969	−0.336	−3.372	0.001	−4.104	−15.920
	Living_alone	−9.260	3.544	−0.259	−2.613	0.011	−16.311	−2.208
	Female	−7.763	3.314	−0.233	−2.343	0.022	−1.170	−14.356

Abbreviations: SE standards error, Sig. significance level.

**Table 4:** Correlation of WHOQOL-BREF domains with the time from the lesion and length of stay.

	WHOQOL-BREF Domains			
	Physical	Psychological	Social	Environment
Time from the lesion (days)	−0.070	−0.087	−0.286 **	0.010
Length of stay (days)	−0.013	−0.195	0.045	−0.097

** Correlation is significant at the 0.01 level (2-tailed).

## Data Availability

The raw data supporting the conclusions of this article will be made available by the authors on request.
